# Optical Quality of InAs/InP Quantum Dots on Distributed Bragg Reflector Emitting at 3rd Telecom Window Grown by Molecular Beam Epitaxy

**DOI:** 10.3390/ma14216270

**Published:** 2021-10-21

**Authors:** Tristan Smołka, Katarzyna Posmyk, Maja Wasiluk, Paweł Wyborski, Michał Gawełczyk, Paweł Mrowiński, Monika Mikulicz, Agata Zielińska, Johann Peter Reithmaier, Anna Musiał, Mohamed Benyoucef

**Affiliations:** 1 Laboratory for Optical Spectroscopy of Nanostructures, Department of Experimental Physics, Wrocław University of Science and Technology, Wybrzeże Wyspiańskiego 27, 50-370 Wrocław, Poland; tristan.smolka@pwr.edu.pl (T.S.); katarzyna.posmyk@student.pwr.edu.pl (K.P.); 245005@student.pwr.edu.pl (M.W.); pawel.wyborski@pwr.edu.pl (P.W.); pawel.mrowinski@pwr.edu.pl (P.M.); monika.mikulicz@pwr.edu.pl (M.M.); 236442@student.pwr.edu.pl (A.Z.); 2 Department of Theoretical Physics, Wrocław University of Science and Technology, Wybrzeże Wyspiańskiego 27, 50-370 Wrocław, Poland; michal.gawelczyk@pwr.edu.pl; 3 Institute of Physics, Faculty of Physics, Astronomy and Informatics, Nicolaus Copernicus University in Toruń, ul. Grudziądzka 5, 87-100 Toruń, Poland; 4 Center for Interdisciplinary Nanostructure Science and Technology (CINSaT), Institute of Nanostructure Technologies and Analytics (INA), University of Kassel, Heinrich-Plett-Str. 40, 34132 Kassel, Germany; jpreith@ina.uni-kassel.de

**Keywords:** MBE growth, symmetric InAs/InP quantum dots, 3rd telecom window, internal quantum efficiency, carrier dynamics, thermal stability of emission, time-correlated single-photon counting

## Abstract

We present an experimental study on the optical quality of InAs/InP quantum dots (QDs). Investigated structures have application relevance due to emission in the 3rd telecommunication window. The nanostructures are grown by ripening-assisted molecular beam epitaxy. This leads to their unique properties, i.e., low spatial density and in-plane shape symmetry. These are advantageous for non-classical light generation for quantum technologies applications. As a measure of the internal quantum efficiency, the discrepancy between calculated and experimentally determined photon extraction efficiency is used. The investigated nanostructures exhibit close to ideal emission efficiency proving their high structural quality. The thermal stability of emission is investigated by means of microphotoluminescence. This allows to determine the maximal operation temperature of the device and reveal the main emission quenching channels. Emission quenching is predominantly caused by the transition of holes and electrons to higher QD’s levels. Additionally, these carriers could further leave the confinement potential via the dense ladder of QD states. Single QD emission is observed up to temperatures of about 100 K, comparable to the best results obtained for epitaxial QDs in this spectral range. The fundamental limit for the emission rate is the excitation radiative lifetime, which spreads from below 0.5 to almost 1.9 ns (GHz operation) without any clear spectral dispersion. Furthermore, carrier dynamics is also determined using time-correlated single-photon counting.

## 1. Introduction

Developing efficient non-classical light sources operating at room temperature and compatible with telecommunication windows is the holy grail of quantum communication and quantum information processing [1,2,3,4,5,6,7,8]. The room temperature operation can be realized in many physical systems [6], including quantum dots (QDs), e.g., GaN-based [9,10], color centers in diamond [11,12,13], defects in SiC [14] or carbon nanotubes [15,16], atoms [17], as well as attenuated lasers [18] and sources relying on the parametric-down conversion process [19,20,21]. Besides QDs and color centers in diamond, all abovementioned approaches address both issues and emit in the telecom wavelengths range [7]. This is important in view of maximizing the transmission distance due to low loss in the transmission channel (both fiber and free space), minimizing the distortion of the optical signal (dispersion), as well as providing compatibility with the existing fiber infrastructure. The key figures of merit for non-classical light sources are, e.g., single-photon purity, photon indistinguishability, and the number of mutually entangled photons. Epitaxial QDs hold performance records in this regards [1,22,23,24,25,26,27,28,29,30,31,32,33,34,35,36,37,38]. These are inherently quantum emitters (with non-classical emission statistics) that have been proven to constitute nearly ideal single-photon sources at emission wavelengths below 1 μm [4,8,38,39,40]. However, using this spectral range is not practical unless a complex, technically demanding, low-efficiency down-conversion process is used [41,42]. There is a proof-of-principle that unprecedented performance can be achieved with QDs [3,6,22,43,44] by optimizing their growth and tailoring their optical properties. Engineering their optical properties can be realized during growth or later by external means, proving their high flexibility. On top of that, they are compatible with the semiconductor technology. Integrated photonic circuits are also developed within this framework. The important milestones achieved with QD structures include the possibility to realize all-optical control while driving them resonantly [45,46,47] and electrical carrier injection [48,49,50,51,52]. The main drawback of these sources operating at telecom spectral range is that their excellent performance can be achieved only at cryogenic temperatures. This is due to a strong increase in the decoherence with temperature due to thermally enhanced interaction with the solid-state environment. At elevated temperatures, carriers escape from the QDs due to finite height of confining potential barriers. It has been proven that single-photon emission can be maintained to temperatures as high as 90 K and 120 K for InP-based emitters in the C-band [50] and GaAs-based ones in the O-band [53], respectively. Unfortunately, the coherent properties of emission are relatively quickly lost with increasing temperature. Therefore, lots of effort is put into developing a compact, cheap, and efficient cooling method to make the QD-based sources practical. This results in commercially available sources emitting below 1 μm [54] as well as laboratory demonstrations of compact devices operating below 1 μm [55] and at telecom wavelengths [56].

**Figure 1:**
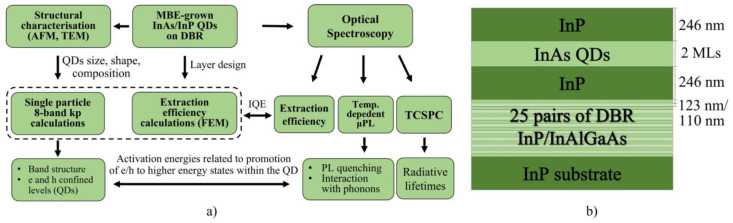
(**a**) Schematic diagram summarizing the steps used in this work (**b**) Scheme of layer design of the investigated structure with InAs/InP QDs on distributed Bragg reflector. Nominal (designed) thicknesses of the respective layers are presented on the right-hand side.

Shifting the emission to the telecom wavelengths is mainly achieved either via strain-engineering of GaAs-based structures [56,57,58,59,60,61,62,63,64,65,66,67,68,69,70,71,72,73,74,75,76,77] or using InAs/InP material system [33,78,79,80,81,82,83,84,85,86,87,88]. In both cases, one of the effects responsible for the redshift of emission is the change in the strain within the heterostructure. This influences the confining potential, and enables growth of larger QDs. However, this results in a smaller interlevel spacing, making the energy structure denser, with more states confined within a QD. For larger structures, the Coulomb interactions are dominant, and they cannot be treated as small perturbations compared to the single particle confinement energy. Hence, transferring the optimized technology for the growth of QDs emitting below 1 μm to telecom wavelength is not straightforward and very challenging. Moreover, modelling the optical properties of these nanostructures might differ from what was developed so far. Therefore, it is necessary to develop new growth methods and investigate the optical properties of such structures. In that way their quality and application potential will be evaluated, providing feedback for further improvement of the epitaxial structures. In this work, we primarily focus on the optical quality of InAs/InP nanostructures. This is quantified by internal quantum efficiency (IQE) determined by comparing the experimental and theoretical photon extraction efficiency data from an as-grown planar structure (Section 3.1). The calculations are performed using commercial implementation (JCMsuite) [89] of finite element method (FEM) for solving time-harmonic Maxwell equations. Experimentally, the emission rate into the first lens was determined based on a count rate on a single-photon detector (SSPD) measured at QD saturation under a non-resonant pulsed excitation. In both, calculations and experimental determination of extraction efficiency, ideal quantum emitter is assumed (IQE of 1). In the case of experimentally determined photon extraction efficiency, this assumption leads to underestimation of the real value. Therefore, the discrepancy between measured and calculated photon extraction efficiency can be treated as a good measure of nanostructures’ optical quality. In our case, it appears to be almost ideal (within the experimental accuracy). Other important aspects in view of both fundamental properties and potential applications, namely carrier dynamics (Section 3.2) and thermal stability of emission (Section 3.3), are also studied experimentally. For that, time-correlated single photon counting and temperature-dependent microphotoluminescence is used, respectively. Study of carrier dynamics focuses on a study of the relaxation processes, excitation lifetimes and influence of the environment on the effective emission decay time. The last one is reflected in differences in lifetime dispersion between planar and patterned QD structure. Temperature-dependent study allows us to identify basic emission quenching channels and evaluate the interaction of carriers confined in the QDs with phonons. It is all done for a novel type of application relevant nanostructures in InAs/InP material system. They provide emission in the 3rd telecom window and demonstrate unique features like low spatial density of the optically active nanostructures, large QD volume, in-plane shape symmetry [85], and high level of As-P intermixing. These result from an additional ripening step during the molecular beam epitaxy (MBE) growth [90,91]. A schematic diagram in Figure 1a summarizes the steps used in this work.

## 2. Materials and Methods

The investigated structure contains a single layer of MBE grown InAs QDs embedded in an InP matrix (246 nm thick InP layer on both sides). QDs are placed on top of the distributed Bragg reflector (DBR). The DBR consists of 25 pairs of InP/In_0.53_Al_0.1_Ga_0.37_As layers of 123 nm and 110 nm nominal thickness, respectively (Figure 1b). The standard epitaxial growth procedure has been modified by adding a ripening step during the QDs growth [90,91]. This results in large in plane QDs with density on the level of 10^9^/cm^2^. The base diameter of the nanoobjects is in the range of (55 ± 15) nm and height-up to 15 nm [92]. More details on the growth are given in Ref. [85]. The spatial QD density is inferred based on atomic force microscopy (AFM) of the QDs grown by the same recipe but without the capping layer [85]. The investigated InAs/InP QDs have previously been tested in view of the single-photon generation and exhibited triggered high-purity single-photon emission in the telecom C-band even under non-resonant excitation [93]. In this work, two structures from the same grown wafer were used: (a) as-grown planar sample and (b) structured sample with cylindrical mesas, which in the text will be referred as patterned sample. The mesa structures were defined on the surface by electron-beam lithography, using SiO_2_ mask and dry-etched with inductively coupled plasma reactive ion etching. The mesas were processed non-deterministically, and the mesa design is not optimized. The purpose of using mesas is to enable repeatable experiments on the same dot and provides the possibility to investigate different aspects of a selected QD in various experimental setups. However, one has to keep in mind that patterning the sample can influence the optical properties of QDs. The relevant effects are change of the photonic confinement and generation of carrier traps on the atomically rough mesa sidewalls. The weak cavity effects can be present in such case due to high refractive index contrast at air/semiconductor interface of the mesa structure. Carrier traps on mesa sidewalls might influence the charge state of the QD, introduce spectral diffusion and nonradiative recombination due to e.g., Auger-type processes. These issues will also be addressed.

For the optical study, two types of experimental setup were used, which are described here. For extraction efficiency and carrier dynamics measurements, a setup based on a NbN superconducting nanowire single-photon detector (SSPD), optimized for telecom wavelengths with quantum efficiency exceeding 80% and time jitter below 50 ps, and less than 100 dark counts/s at 1550 nm, was utilized. As this is a single-channel detector, the optical signal is first filtered spectrally with a 0.55 m focal length monochromator. It provides 1.4 nm bandwidth (compared to the typical QD emission peak linewidth of 0.2 nm) sufficient to isolate a single emission line from the QD spectrum. The signal is further processed with a multichannel picosecond event timer featuring time-bin widths down to 4 ps. The emission decay times are measured using a time-correlated single-photon counting technique. The non-resonant excitation is provided by a semiconductor pulsed laser diode at 805 nm, with pulse length below 50 ps at 80 MHz repetition rate. The overall temporal resolution of the experimental setup is on the level of 80 ps. The thermal stability study was performed in a microphotoluminescence (μPL) setup. The setup features 20 μeV spectral resolution. It is equipped with a 1 m focal length monochromator combined with an InGaAs multichannel detector. In this setup, continuous-wave (cw) non-resonant excitation with a 640 nm semiconductor laser was used. All the measurements were performed at 4.5 K in a continuous flow liquid He micro-cryostat. The required spatial resolution is provided by long working distance (20 mm) achromatic infinity-corrected microscope objective with 0.4 numerical aperture. It is diffraction-limited to an approx. 2 μm laser spot diameter on the sample surface.

The experimental results are compared with theoretical calculations concerning two aspects. At first, the extraction efficiency is calculated using a commercially available implementation of the finite element method—JCMwave software [89]. The Maxwell equations are solved for the given design and layer structure with perfectly matched layer boundary conditions. Next, the electromagnetic field distribution inside the structure due to QD emission is calculated. The numerical box has dimensions of 4 μm width and 6.75 μm height to avoid artificial photonic confinement and modes. To obtain more realistic input parameters for the thicknesses of the DBR layers, low temperature reflectivity spectra measured on the planar sample and simulated within transfer matrix approach [94,95,96] (not shown here) were compared. The actual thicknesses of 124.8 nm and 111.3 nm for InP and InAlGaAs, respectively, were determined. The final layer thicknesses used in the extraction efficiency calculations were adjusted so that the wavelength of the maximal photon extraction efficiency in the experiment and theory matches. This is achieved for the thickness of InP and InGaAlAs layers equal to 125.5 nm and 111.5 nm, respectively. It was carefully checked that the exact thicknesses of the DBR layers do not influence the maximal achievable photon extraction efficiency (not shown here). This is the figure of merit in evaluating the optical quality of the investigated nanostructures. It only shifts the emission wavelength dependence of the photon extraction efficiency horizontally. For the photon extraction calculations, the refractive indices for InP and InGaAlAs are taken to be 3.13 and 3.38 at low temperature. These are obtained from the fitting to the low temperature DBR reflectivity spectrum. They agree well with the values reported in the literature [97,98,99,100], including interpolation in the case of InGaAlAs material. The QD itself is modeled as a point dipole source. This assumes that details of the nanostructure size and shape are not crucial for the electromagnetic field distribution in the far field. The extraction efficiency is calculated as a ratio between the power emitted by the dipole into the given solid angle and the total emitted power. For comparison with the experiment, a solid angle corresponding to 0.4 numerical aperture of the collection optics of the experimental setup is used. More details on the simulation approach can be found in Ref. [93].

The calculations of the confined states energy structure of the exemplary QD were performed in the single particle-picture using the eight-band ***k∙p*** method [101,102]. These include realistic strain distribution calculated within the continuous elasticity approach and piezoelectric effect up to the second-order in strain tensor components. They were performed to support the interpretation of the temperature-dependent μPL results. QD parameters, namely the diameter of 42 nm and height of 15 nm (lens-shape), are used in the calculations. The QD is placed on top of a 1.2 nm thick wetting layer and embedded in an InP matrix. For such a large purely InAs QD, the emission energy appears far too low. Therefore, a high level of As-P intermixing (25%) was included to bring the calculated ground state energy in agreement with the experimental findings. Measured emission from the QD ensemble at 4.5 K is centered at 0.805 eV. For simplicity and due to the lack of statistically relevant data in this regard, a homogeneous P distribution was assumed. The exact material parameters and more details on the modeling can be found in Ref. [88]. The implementation of the used ***k∙p*** method is described in [103].

## 3. Results

### 3.1. Internal Quantum Efficiency

The internal quantum efficiency of an emitter is an essential quantitative figure of merit when optical quality is considered. It reflects the structural material quality and evaluates indirectly how well are the growth and fabrication procedure optimized. However, it is not easy to directly measure this quantity for embedded QDs [104,105] emitting in the infrared range and at low temperatures. In most cases, the emission intensity is measured relative to a reference structure or previous generation of nanostructures of interest. Below, the optical quality is quantified by comparing measured and calculated photon extraction efficiency from a planar structure. The justification of this method is based on the assumption that the main difference between the calculated and measured extraction efficiencies originates from non-ideal internal quantum efficiency. The planar sample is used since this is the simplest setting realized in practice. Including photonic structures makes the system more complex. Therefore, other sources of discrepancy between results of calculations and measurements might appear. We want to remain in the regime where the internal quantum efficiency is the dominant factor. It should be noted that in both, the calculations and the experiment, 100% internal quantum efficiency is assumed. If the calculation results match the experimental value, it can be deduced that one deals with an almost perfect emitter. In the experiment, the number of photons collected by the first lens in the optical setup per second is divided by the repetition rate of the excitation laser. It is therefore assumed that each excitation pulse leads to carriers being captured in the QD and one electron-hole pair per pulse is recombining radiatively. The former is related to the excitation efficiency. To make it close to unity, i.e., to assure the occupation of the QD by one electron-hole pair per pulse, the saturation excitation power is used. The latter depends on the internal quantum efficiency of the emitter and is limited by the nonradiative recombination channels. The measurements are performed at cryogenic temperatures to minimize decreasing the number of emitted photons by temperature-dependent PL quenching mechanisms (Section 3.3). There are two other effects that one has to avoid: exciting the same state of a QD twice within the duration of the excitation pulse and higher excitonic complexes starting the cascaded emission. Both lead to more than a single photon emitted from a QD per pulse. For example, generation of the biexciton in a QD will lead to emission of two photons—one from a biexciton generated via optical excitation and the second one from an exciton left after the recombination of the biexciton, i.e., not originating directly from the optical excitation. The first issue can be minimized by choosing the proper pulse length—at least one order of magnitude shorter than the PL lifetime. Therefore, we used <50 ps long pulses in comparison to the shortest measured lifetimes on the level of 0.47 ns. To exclude cascaded emission events, neutral or charged excitons, i.e., those with up to three carriers confined in the QD, are only of interest. Therefore, we focus on emission lines present in the optical spectrum for low excitation powers. More complicated excitonic complexes require higher excitation powers, providing a high enough probability to capture more than two carriers in the QD. On the other hand, if there is a non-negligible probability of capturing both exciton or trion in the QD, the correct procedure for determining extraction efficiency would be to sum up emission intensity from these two complexes. They are mutually exclusive (either exciton or trion is formed and recombines for a given excitation pulse). Thus, it does not lead to artificial multiplication of the number of photons emitted per pulse. In that case, the determination of extraction efficiency based on a single emission line would lead to its underestimation. This could be another source of discrepancy between theoretical and experimental results.

Having all this in mind, we performed the following experiment. We investigated five different spots on the planar sample surface. For each of them, we measured low and high excitation (saturation power) spectra on the SSPD detector under non-resonant pulsed excitation. For further determination of the extraction efficiency, the highest intensity emission lines taken at low excitation powers are selected. From them, emission lines present in the spectrum already for low excitation are of interest, as discussed above. The experimental extraction efficiency was determined using the following formula:

(1) $$\eta_{extraction}=\frac{n_{SSPD}}{f_{Laser\;}\eta_{Setup}}\;$$

where *n_SSPD_* is the count rate measured on the single-photon detector at saturation power, *f_laser_* is the repetition rate of the excitation laser, and *η_setup_* is the efficiency/transmission of the experimental setup. This efficiency is determined in an independent experiment. For that purpose, the transmission of each optical element in the setup was measured for a laser with a wavelength corresponding to the QD emission. The quantum efficiency of the detectors measured during their installation (this includes the transmission of the fiber attached to the detectors and coupling of the optical signal from the fiber to the detectors) was considered. The setup efficiency is then the product of transmission of all the elements in the setup, which in our case yields 1.15%.

**Figure 2:**
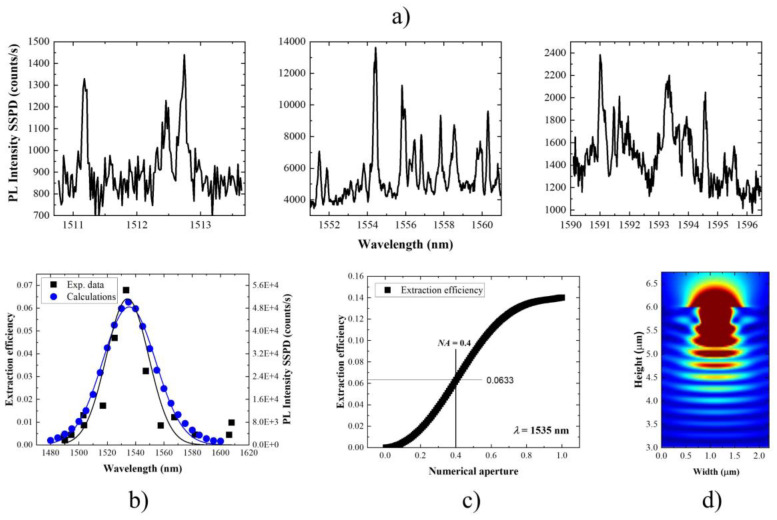
(**a**) Exemplary photoluminescence spectra used for extraction efficiency measurements (non-resonant pulsed excitation with power corresponding to saturation conditions) (**b**) Experimental (black squares) and calculated (blue dots) extraction efficiency for NA = 0.4 (left axis) with Gaussian fits (solid lines); right axis–PL intensity under non-resonant pulsed excitation with saturation power measured on SSPD at 5 K (whole spectrum on Figure 3; (**c**) calculated extraction efficiency vs. numerical aperture of the first lens collecting the optical signal for source wavelength of 1535 nm for which maximal extraction efficiency was obtained; NA = 0.4 corresponding to experimental conditions marked with solid black lines; (**d**) calculated distribution of electromagnetic field for 1535 nm wavelength, for which maximal extraction efficiency was obtained; the intensity of electric field component is color-coded and presented in a logarithmic scale; the calculations are performed for numerical aperture of 0.4.

The extraction efficiency was experimentally determined for 15 emission lines of various emission wavelengths. Its dependence on the emission wavelength is presented in Figure 2b. It spans from 0.2% to 6.8% (left axis/scale) corresponding to 56.5 kHz of maximal emission rate measured on SSPD (right axis/scale). Clear dependence on the emission wavelength can be observed. This is well-reproduced by a Gaussian profile centered at 1533 nm and 35 nm wide.

Theoretically obtained extraction efficiency gives qualitatively and quantitatively similar dependence on emission wavelength (Figure 2b). The maximal extraction efficiency yields 6.3%, which is equal to the maximal experimental value within the experimental accuracy. The uncertainty is estimated to be on the level of 1%. Division of the experimental worst-case scenario (the difference between experimentally obtained extraction efficiency value and its accuracy yielding 5.8%) and the theoretical limit yields 92% internal quantum efficiency. This shows that the investigated QDs in a planar sample are almost ideal quantum emitters. This means that the emission is not significantly disturbed by nonradiative processes. Such high IQE reflects the high structural quality of the epitaxial material. This value can be used as a correction factor when extraction efficiency is determined for QDs in a more complex photonic environment. Instead of assuming 100% internal quantum efficiency, which leads to underestimated values, one can include the experimental estimation obtained above. The theoretical dependence on emission wavelength has a larger broadening than the experimental one—44 nm compared to 35 nm, respectively. Theoretical emission wavelength dependence is governed only by the photonic environment. This includes DBR spectral characteristics and the effect of low finesse cavity (formed between the DBR and the sample surface/air interface). The IQE itself equals 1 for all the wavelengths. In the case of the experiment, wavelength dependence is additionally influenced by the IQE and by the dot-to-dot differences. The experimental extraction efficiency is determined based on a single high-intensity emission line. In fact, this constitutes the lower limit of the actual photon extraction efficiency (and, as a result, the upper limit of the IQE). This is because it is only accurate in the case when there is one dominating charge state formed in the QD. This implies that the probability of emission via other channels is negligible. In an extreme case in which the probability of formation of a neutral exciton and a trion is comparable, the measured value would be only half of the actual one. This could explain dot-to-dot differences in the determined figure of merit and discrepancies with the results of the calculations. In the case of investigated QDs, it is a complex matter to distinguish between neutral exciton and trions due to ultra-low fine structure splitting, below the resolution of the standard experimental setup [92]. Therefore, we did not explore this topic further. It would require complex experimental methods, including the application of external fields and sample processing (fabricating markers on the sample surface), to be able to investigate the same QD in a planar structure repeatedly. This, however, does not limit the validity of the estimated IQE. For a vast number of nanostructure groups, this is rather straightforward to identify excitonic complexes from the same QD by means of excitation power-dependent and polarization-resolved μPL measurements. In that case, the IQE can be determined with higher accuracy. In our case, we focus on the maximal achieved value of photon extraction efficiency, as only this has a clear interpretation.

To get more insight into the directionality of the emission from the investigated nanostructures, extraction efficiency was calculated as a function of NA of the detection optics (Figure 2c). The photon extraction efficiency for NA equal to 1 yields a value of 14%. This is one order of magnitude higher than in the case of a QD embedded in a homogeneous semiconductor medium [67,106]. This shows the importance of the bottom DBR section for increasing the extraction efficiency. It is consistent with one order of magnitude improvement in emission intensity of the QD ensemble in comparison to the structure without DBR [85]. The calculated electromagnetic field distribution for the experimental NA and wavelength corresponding to the maximal photon extraction efficiency value is presented in Figure 2d. It shows that the number of DBR pairs is sufficient to minimize the emission into the substrate. Additionally, there are no substantial photon losses in the sample plane. However, the emission in the far field is not strongly directional, as expected for a planar structure. To obtain higher photon extraction efficiency, the emission would need to be made more directional. This can be realized either by adding a top DBR section and forming a microcavity. The drawback will be a narrow-band operation of such a source. In that case, fulfilling the resonance condition between the emitter and the cavity mode is required. The other possibility is patterning of the sample surface, preferably in deterministic fashion. Note that 13.2% photon extraction efficiency has already been achieved using non-deterministic mesas of optimized design fabrication [107]. This is still a factor of 2 lower than the value theoretically predicted [108]. This suggests that the patterning of the sample might influence the IQE, which lowers the expected gain in the photon extraction efficiency.

### 3.2. Carrier Dynamics

Carrier dynamics was investigated in terms of time-correlated single-photon counting. Each emission line had to be measured separately. All the measurements were performed at low temperature (4.5 K) and low excitation. It was done to minimize the influence of the nonradiative recombination channels in order to be able to access the radiative lifetime. For higher excitations, the relaxation processes might influence the measured PL decay curves. As a result, an effective decay time, instead of the radiative recombination time, is measured. The measurements are performed on both the planar and patterned samples, to investigate the influence of the sample patterning on the carrier dynamics.

**Figure 3:**
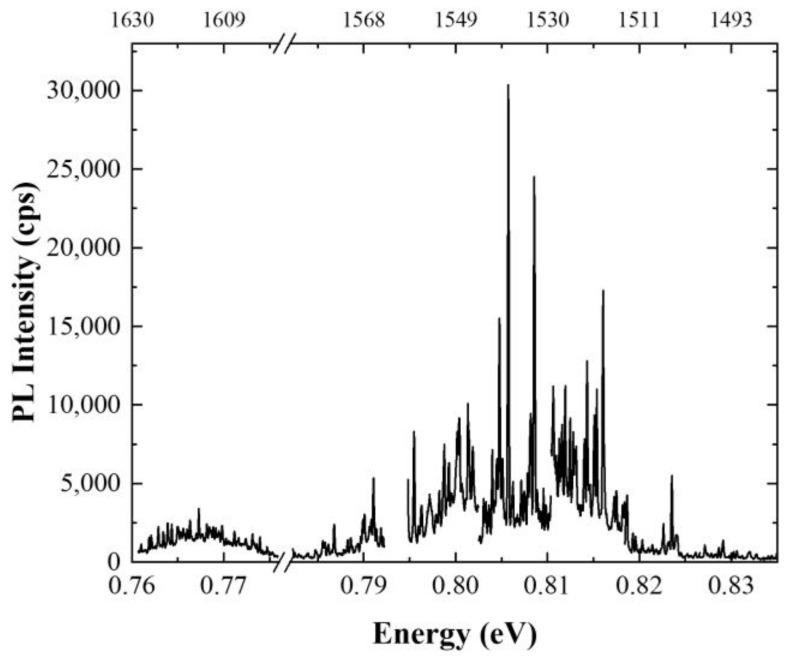
Low-temperature (T = 4.5 K) photoluminescence (PL) spectrum from planar Scheme 0. (μW) using single-photon superconducting nanowire detectors.

**Figure 4:**
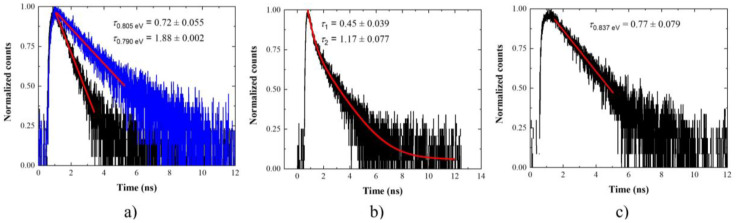
Normalized low-temperature (T = 4.5 K) and low-excitation time traces of exemplary single emission lines from the planar structure with InAs/InP QDs showing distinct behavior: (**a**) mono-exponential decay with much different PL decay times; (**b**) two-component exponential decay; (**c**) decay with initial rise time (0.08 ns) together with linear fits to experimental data.

Figure 3 presents an exemplary single-channel PL spectrum for planar structure at low excitation powers. The spectra at different positions on the sample were measured, covering in total approx. 75 meV (almost 150 nm) spectral range. This covers the 3rd telecommunication window. The decay curves were measured and analyzed for 90 emission lines. The measured curves were fitted with either mono- or two-component exponential decay. The characteristic PL decay times were determined from this fitting procedure. The uncertainties of the PL decay time given in the graphs are determined solely from the fitting procedure. These do not include the experimental accuracies. In many cases, it seems that there might be another long-timescale process present in the experimental data. This has not been investigated in detail as we are mostly interested in the fastest processes. This one sets the fundamental limit for generation rate from investigated nanostructures. In Figure 4, exemplary PL decay curves measured on the planar sample are presented. Three types of behavior can be distinguished: (i) mono-exponential decay (Figure 4a) observed for 92% of all the investigated emission lines, with PL decay times in the range of (0.72–1.88) ns; (ii) two-component exponential decay—8% of cases (Figure 4b), with the faster component τ_1_ in the range of (0.45–1.33) ns and the slower component τ_2_ in the range of (1.06–1.95) ns and positive correlation between the two lifetimes; (iii) measurable rise time up to 0.15 ns followed by either mono- or two-component exponential decay—15% of cases (Figure 4c). As can be seen, the mono-exponential decay is by far the most typical behavior for the investigated structure. From the energy dispersion it seems it has no clear emission energy dependence. However, quite a broad distribution of the PL decay times is observed for a given emission energy. This might result from different excitonic complexes being analyzed, because identification of the origin of the emission lines was not performed. Flat lifetime dispersion was previously observed for nanostructures featuring an intermediate confinement regime. This is expected for large QDs [109]. It can also be a fingerprint that the emission energy is not determined by a single parameter. For example, both material composition and size differ from dot to dot. In that case, the emission energy changes, but the oscillator strength, and so the lifetime, is not strongly influenced. The limited structural data is insufficient to support the latter hypothesis. Observation of the PL rise time hints at the presence of an intermediate state in the relaxation path of the carriers created in the substrate material (non-resonant excitation). This can be the case, e.g., for a defect state in the vicinity of the QD effectively capturing the carriers on their way to QDs. Alternative might be a biexciton–exciton cascade. Recombination of the biexciton results in occupation of the exciton. Therefore, the PL rise time, corresponding to the lifetime of a biexciton, occurs in the PL time trace. This happens for excitation powers high enough to ensure occupation of the biexciton state [110]. In this case, the emission line which is analyzed is not occupied directly from the optical excitation, but some intermediate state within or in the vicinity of a QD mediates its occupation. The main relaxation mechanism in the case of non-resonant excitation–phonon relaxation, leads to the occupation of a given state in a femtosecond to single picosecond time scale [111]. This is too fast to be resolved in this experiment. The rise time is seen even for a low excitation power. In this excitation conditions, the probability of forming multicarrier complexes should be negligible. This suggests that it is rather not related to cascaded emission within the QD. The two-component exponential decay is not typical for investigated structures, as it is rarely observed. Similar decay curves, with a dispersion-less fast component, were previously observed in strongly asymmetric quantum dashes in InAs/InGaAlAs/InP. The two components were interpreted as corresponding to two bright excitons differing strongly in the transition oscillator strength [112]. In our case, the structures are symmetric, and therefore the same mechanism cannot be responsible for the substantial difference in lifetime between the two transitions. However, a significant degree of linear polarization of up to 50% was observed for single emission lines for these QDs (not shown here). This would be enough to explain the observed timescale differences. The gathered experimental data does not allow for unambiguous interpretation of the second PL decay time component, but this is something to keep in mind if fast QD depopulation is of interest.

**Figure 5:**
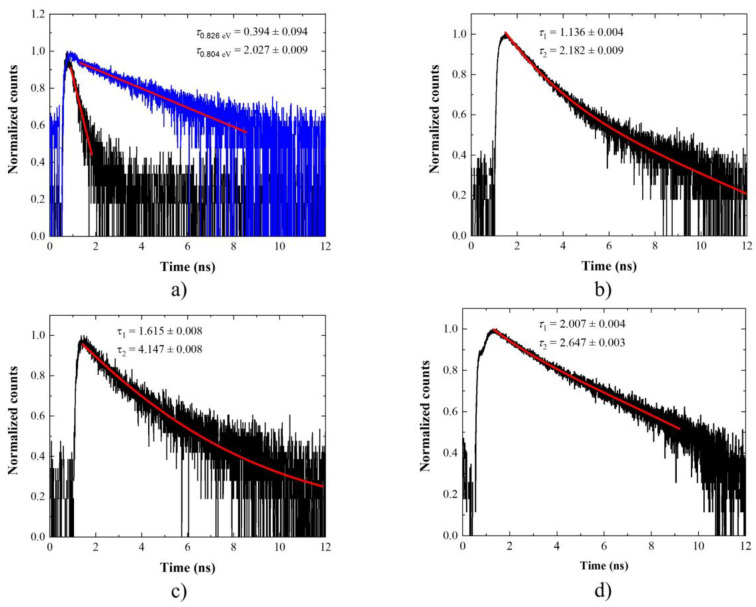
Normalized low-temperature (T = 4.5 K) and low-excitation time traces of exemplary single emission lines from patterned structure with InAs/InP QDs showing distinct behavior: (**a**) mono-exponential decay with much different PL decay times; (**b**,**c**) two-component exponential decay; (**d**) decay with initial rise time together with exponential fits to experimental data.

**Figure 6:**
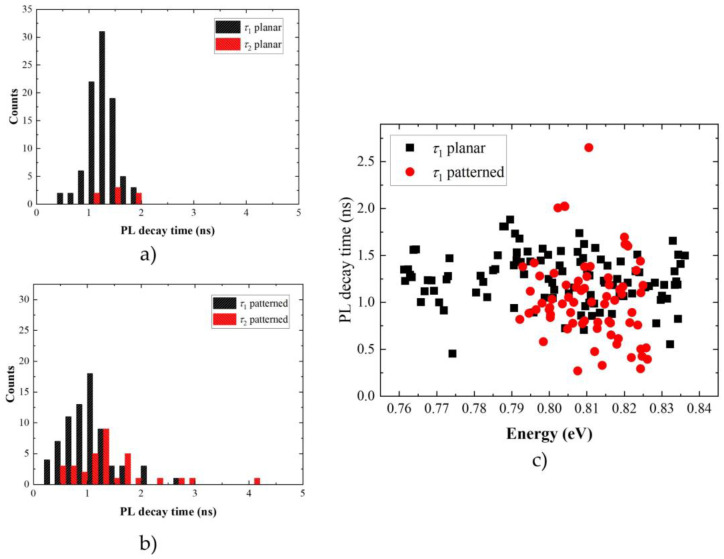
Carrier lifetime distribution for (**a**) planar and (**b**) patterned sample with initial PL decay time τ_1_ marked in black and longer PL decay time τ_2_ marked in red. (**c**) PL decay lifetime τ_1_ dispersion for planar (black) and patterned (red) sample.

A similar study for 72 emission lines in 4 mesas was performed on the patterned sample. Similar types of behavior were observed (Figure 5). This time mono-exponential decay with a characteristic lifetime in the range of (0.39–2.65) ns was measured for 54% of emission lines. Two-component exponential decay with fast and slow components in the range of (0.27–2.01) ns and (0.52–4.15) ns, respectively, for 46% of cases. Again, positive correlation between the two times was seen. A measurable rise time was detected for 21% of the cases, with the rise times up to 0.13 ns. This seems to be similar for planar and patterned sample. The main difference is much larger contribution of the cases with a second PL decay time component. As far as the lifetime dispersion is considered (Figure 6c), it is clear that a subensemble of QDs, corresponding to a narrower emission range, was investigated. There is no clear energy dependence of the PL decay lifetime, but the distribution of lifetimes for a given energy is much broader than in the case of a planar structure. This difference might be related to the fact that other excitonic complexes are allowed in the case of the patterned sample. Excitonic complexes can differ substantially in the radiative lifetime. Some of them might not be present or at least not measurable in the case of the planar sample. Different dominant charge state might arise from, e.g., roughness in mesa sidewalls acting as charge traps in the vicinity of QDs. These might deplete or support a given type of carrier, increasing the probability of forming charge excitonic complexes. For the case of the investigated QDs in mesa structure, it was shown that the emission is of mainly charged character [92]. Similar studies were not carried out for the planar structure. This would require combination of a complex experimental methods and the need for repeating various experiments on the same QD. This was not possible for the planar structure. Additional carriers that can be trapped on the etched mesa sidewalls provide a charge environment with stronger fluctuations closer to the QD. Additional carriers facilitate the formation of charged excitonic complexes in the QDs and this will depend on the position of the QD. These effects are of random character and not directly related to the energy spectrum of a QD and therefore not directly linked to their emission energy. The reduction of the PL decay lifetime might also result from a nonradiative recombination channel (e.g., Auger-type processes with carriers trapped on mesa sidewalls) or Purcell enhancement of the emission in the case of mesa photonic structure [108]. In the case of random position of the QD in the mesa structure due to the non-deterministric fabrication processs, it is also possible to extend the PL decay lifetime (Purcell factor is lower than 1).

For the sake of comparison between the patterned and planar sample, histograms of both PL decay time components are summarized in Figure 6a,b. The lifetime distribution is narrower and centered at longer lifetimes for the planar sample, with rare cases of the second slow time component. In the case of the patterned sample, the longer time component appears frequently. The distribution of both the PL decay times is broader compared to the planar sample. Additionally, their mean value is shifted towards shorter times. This agrees with adding a random effect leading to stronger differentiation of the dynamics between the dots. It can be related to mesa sidewalls, the distance of which to the investigated QD is random. Other excitonic complexes can possible be formed, thanks to carrier exchange with traps in the vicinity of QDs. The second, slow time component, prevents fast deexcitation of a given QD state. This is detrimental for many applications. Obtained results point at the necessity for deterministic positioning of the QD within a mesa and for optimizing the mesa fabrication to maintain the optical quality of investigated QDs in photonic structures.

### 3.3. Thermal Stability of Emission

**Figure 7:**
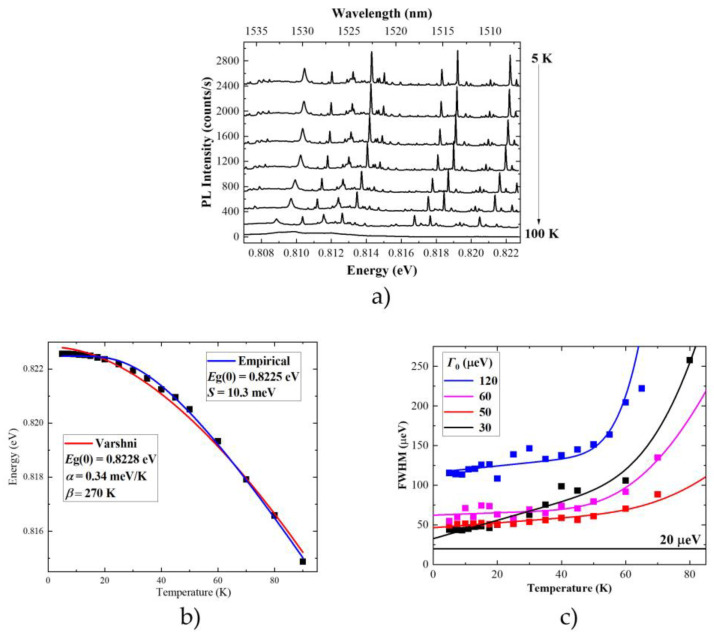
(**a**) Temperature series (5–100) K of microphotoluminescence spectra under non-resonant cw excitation with 2 μW. (**b**) Emission energy of exemplary emission line with respect to the temperature determined from Gaussian fit to experimental data (black squares) fitted with Varshni formula [115] (solid red line) and formula derived from microscopic description of the exciton-phonon interaction (Equation (2), solid blue line) with respective fitting parameters. (**c**) Examples of typical behavior for full width at half maximum (FWHM) of the emission line with respect to the temperature determined from Gaussian fit to experimental data (symbols) fitted with the formula [116] Equation (3) for emission lines with initial FWHM (at the temperature of 5 K) of 30 (black), 50 (red), 60 (pink), and 120 μeV (blue). The spectral resolution of the experimental setup (20 μeV) is marked with a black horizontal line.

The thermal stability of emission is another important figure of merit when evaluating the application potential of quantum emitters. The main issues to be considered are how strong is the influence of phonons on the emission and up to which temperature it is possible to detect emission from single QDs. Study of the emission intensity as a function of temperature typically allows identifying the processes responsible for the PL quenching. This helps to find means necessary to improve their performance. To study the thermal stability of emission from the investigated nanostructure, 15 single emission lines in 3 different mesas were measured. A temperature series were recorded and analyzed. Emission from single QDs is observed for temperatures in the range of 100 K, comparable to 80 K obtained in this spectral range for InAs/GaAs QDs on a metamorphic buffer layer [113], InAs/InGaAlAs/InP quantum dashes [114] and low-density InAs/InP QDs [88], as well as 100 K for InAs/InP quantum-LED [50]. For all the emission lines, typical thermally-induced behavior of the emission spectra is observed: a systematic redshift of the emission energy, linewidth broadening, and monotonic decrease of emission intensity (exemplary spectra presented in Figure 7a). All these aspects are analyzed and interpreted in detail below. The determined parameters are summarized in Table 1.

All the parameters (emission energy, FWHM, and intensity) are determined from a Gaussian fit to experimental data performed for individual emission lines. Emission intensity is evaluated in terms of integrated intensity (peak area), so the change in the lineshape (its broadening) is included. The emission energy for all the lines is almost constant up to 20 K, and then it decreases strongly. The main effect is related to the temperature dependence of the semiconductor bandgap energy. It originates from thermal expansion of the crystal lattice and the interaction of optical excitations with phonons. Fitting this dependence with the phenomenological Varshni formula shows discrepancies in the low and intermediate temperature range. This is typical for the low-dimensional structures [98] and points out that the changes in the energy bandgap are not the only effect influencing the emission energy. The determined α parameters are in the range of (0.24–0.38) meV/K (Table 1). These are in between the values known for bulk InAs (0.276 meV/K) and InP (0.363 meV/K) [117], respectively. This suggests that As-P intermixing is present in the investigated nanostructures. However, no quantitative conclusions can be derived based on these measurements, as the experimental uncertainty is too large. Also, no clear dependence between the α parameter and the emission energy can be observed. This suggests that the composition of the QD material is not solely determining the emission energy. However, again, the large experimental uncertainty might hinder the actual dependence. The β parameter is much larger than in bulk material, which is typical for systems with lower dimensionality [118]. It is also not very reliable when the emission cannot be observed up to the room temperature. One has to keep in mind that difference in the thermal expansion of different materials within the heterostructure influence the depth of confining potential. This changes the single-particle levels, therefore, changing the emission energy on top of the band edge variation. Microscopic theories providing descriptions of the bandgap energy changes with temperature based on analysis of the electron-phonon interactions allow for better agreement between the data and the fitted curve. This shows that observed dependences can be reproduced if interaction with phonons is described properly (Figure 7b). The experimental data were fitted with the following formula [119]:

(2) $$E_{g}\left(T\right)=E_{g}\left(0\right)-S\langle \hbar \omega \rangle \left[coth\left(\frac{\langle \hbar \omega \rangle }{2k_{B}T}\right)-1\right],\;$$

where $E_{g}\left(0\right)$ is the bandgap at zero temperature, S is a dimensionless exciton–phonon coupling constant, 〈ℏω〉 corresponds to the average phonon energy and $k_{B}$ is Boltzmann constant. Determined phonon energies (Table 1) point out that the phonons in a bulk matrix material (InP) are mainly contributing to the change of the emission energy [120]. The obtained exciton–phonon coupling constant is in the range of 0.92 to 1.3. The interaction with phonons can also be probed by the linewidth of emission. Exemplary data are presented in Figure 7c. Low-temperature linewidth is in the range of (30–120) μeV (line-to-line differences). These values are up to few times lower than in the case of MOVPE-grown low density InAs/InP QDs [88] and strongly asymmetric compared to InAs/InGaAlAs/InP quantum dashes [121,122] and comparable to the state-of-the-art GaAs-based QDs grown on metamorphic buffer layer [123] (all emitting in the 3rd telecom window). All these values exceed the spectral resolution of the experimental setup (20 μeV) and are much larger than Fourier-transform limited linewidth for lifetimes presented in the previous section (corresponding to 1.5 μeV at maximum). Therefore, we conclude that spectral diffusion affects the measured spectral linewidth. The differences in low-temperature linewidths can be caused by different charge environments of a particular QD or by different excitonic complexes confined in the same QD. The former leads to larger amplitude of spectral wandering. Due to differences in wave function distribution and polarizability, the susceptibility of the emission energy of the specific exciton complex to the electric field effect differs [124,125]. With increasing temperature, the broadening remains almost constant up till approx. 50 K and then increases abruptly. This is consistent with the temperature-dependent broadening caused by higher phonon states occupation and stronger exciton-phonon interaction [126]:

(3) $$\Gamma =\Gamma_{0}+\gamma_{Ac}T+\gamma_{LO}exp\left(-\frac{\Delta E}{k_{B}T}\right),\;$$

where $\Gamma_{0}$ is a zero-temperature linewidth, $\gamma_{Ac}/\gamma_{LO}$ are acoustic/optical-phonon broadening efficiency and ΔE corresponds to the energy of longitudinal optical (LO) phonon coupled to the exciton. The exact shape of this relation depends on the strength of the spectral diffusion effects and on the level at which the acoustic phonon-related sidebands contribute to the total emission. If their contribution is dominant, the FWHM is no longer determined by the zero-phonon line (ZPL) broadening [114]. From the fit to the experimental data, the broadening efficiency related to both types of phonons and the characteristic energy of the optical phonons in the range of (14–86) meV were determined. Similar values were previously reported for other InP-based nanostructures, even with much different barrier material, e.g., self-assembled InAs/Ga_0.2_In_0.8_As_0.4_P_0.6_/InP quantum dashes [127]. This indicates that phonon modes of interest are related to the InP matrix. The LO phonon energy of bulk InP of 30 meV lies within this range [128]. The large distribution of obtained values is related to the fact that in the experiment, broadening does not originate only from the phonon effects but also from the spectral diffusion. The spectral diffusion is also temperature-dependent. Additionally, the figure of merit is the FWHM of the total emission (sum of the ZPL and acoustic phonon sidebands) [114]. Also, it is not without consequence that the emission can be observed on average up to 100 K, at which the LO phonon interactions become important.

**Figure 8:**
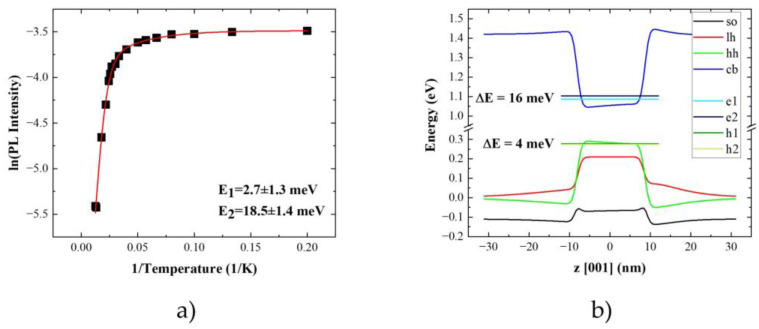
(**a**) Arrhenius analysis (solid red line is a fit with Equation (1)) of temperature dependence of the integral emission intensity determined from Gaussian fit to experimental data (black squares) for exemplary emission line. (**b**) Calculated single-particle energy spectrum (ground state-1 and first excited state-2) for electrons (e) and heavy holes (hh) with the band edges for conduction band (cb) and hole subbands (lh-light holes, so-spin-orbit split-off subband) for the typical QD (42 nm base diameter, 15 nm height, 25% P content), with energy difference between ground state single-particle levels (e1–h1) equal to 0.810 eV (1531 nm).

The last analyzed observable is the emission intensity. The typical behavior, together with Arrhenius analysis, is presented in Figure 8a.

**Figure 9:**
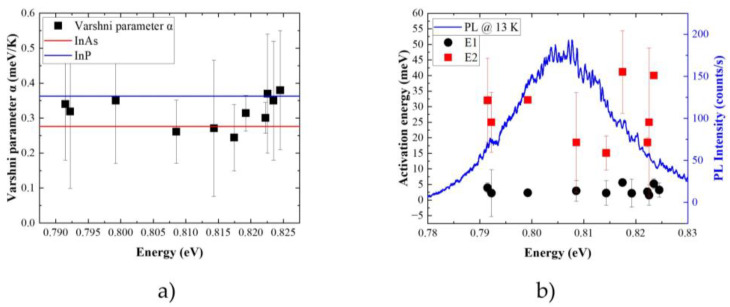
(**a**) Emission energy dependence of the Varshni parameter α for emission lines investigated on the patterned sample determined from a fit to experimental data (black squares with error bars corresponding to the fitting accuracy); theoretical values for bulk InAs and InP are marked with the red and blue horizontal line, respectively; (**b**) activation energies as a function of QD emission energy (symbols, error bars correspond to the fitting accuracy, left axis) with low-temperature (13 K) PL spectra of the QD ensemble (solid blue line, right axis).

The PL quenching data was fitted with a standard Arrhenius formula, including two activation energies [129]:

(4) $$I\left(T\right)=\frac{I_{0}}{1+\sum_{i=1}^{N}C_{i}exp\left(-\frac{Ei}{k_{B}T}\right)}$$

where $I_{0}$ is the initial intensity at low temperature and N is the number of quenching mechanisms. The activation energy (or energies) together with efficiencies *C_i_* of the respective processes are determined. These activation energies are around 25 meV and 3 meV for the higher and the lower efficiency process, respectively (Figure 8a). These are way too small for the carrier escape to the wetting layer or InP matrix directly. Therefore, they are interpreted as the carrier escape via the higher energy states within the QD. This is highly probable as the QDs are large, and the ladder of states is dense, so many states are confined within the QD. To get a deeper insight into the interpretation of determined activation energies, a single-particle energy spectrum of an exemplary QD was calculated. QD with a ground state transition corresponding to the energy of maximal PL intensity of the QDs’ ensemble was selected as the representative one. The results of the calculations are presented in Figure 8b. The *s*-*p* splitting equals to 20 meV and is shared in the relation 1:4 between the valence and conduction band, respectively. This allows us to interpret the lower/higher activation energy as a promotion of holes/electrons to the higher states within the QD. The determined activation energies do not show a clear dependence on the QDs’ emission energy (Figure 9b). This could be related to the fact that investigated examples are within a rather narrow energy range of 30 meV. It might be too small to reveal emission energy trends in the s-p splitting. Also, the dense energy spectrum can lead to a lack of dependence. Activation to different closely separated states is possible. Similar activation energies for PL quenching processes were previously reported for other InP-based structures emitting at 1.55 μm, i.e., low density InAs/InP quantum-dot-like structures grown by MOVPE [88] and MBE-grown InAs/InGaAlAs/InP QDs [90] as well as strongly elongated quantum dashes [122,130]. In the former, it was interpreted as a carrier escape to the excited state within QD alike our case. The investigated structures are also relatively large, with the height exceeding 9 nm. For quantum dashes, 20 meV was already enough for the electrons to escape directly to the wetting layer.

**Table 1:** Summary of the parameters determined from temperature-dependent microphotoluminescence measurements.

Parameter	Experiment	Literature	
α	(0.24−0.38) meV/K	InAs: 0.276 meV/K InP: 0.363 meV/K	[117]
β	(161−340) K	InAs: 93 K; InP: 162 K	[117]
S	(0.92−1.30)		
〈ℏω〉	(9.2−12.6) meV	2.07–23.99 meV	[120]
$\gamma_{Ac}$	(0.03−1.65) μeV/K		
$\gamma_{LO}$	(1.2−105.2) meV		
ΔE	(14−86) meV	29.77 meV	[128]
$E_{1}$	(1.6−5.7) meV		
$E_{2}$	(15.1−41.0) meV		

## 4. Conclusions

In conclusion, we investigated the optical quality of application relevant MBE grown InAs/InP QDs emitting at the telecom C-band. The IQE was estimated by comparing the experimentally and theoretically determined photon extraction efficiency for QDs in a planar sample. The lower limit of maximal IQE for investigated nanostructures in a planar sample is 92%, which reflects the high optical quality of the grown structure. The thermal stability of QD emission was also investigated. Single QD emission was observed up to 100 K, which is comparable to the best results reported in this spectral range. Comparing the activation energies determined from the Arrhenius analysis and eight-band ***k·p*** calculations of the single-particle energy spectrum allows us to identify the main cause of the PL quenching. That is the activation of electrons and holes to higher-energy states within the QD due to closely spaced energy levels, which is expected for large nanostructures. Improving the temperature stability of emission would require engineering of structures towards smaller heights. This would result in increasing the energy separation within the electron and hole ladder of states. The study on carrier dynamics revealed characteristic lifetimes, allowing in principle for GHz operation of QD-based devices. The obtained data from the patterned sample show a broader distribution of lifetimes and more complex dynamics with a second (longer) component present for most of the emission lines. To gain better understanding, further improvement of the fabrication process is required. To maintain the high optical quality of investigated QDs in photonic structures, optimization of the mesa design as well as deterministic positioning technology are required. These will allow for maximizing the emission extraction efficiency by matching to the optical properties of single QDs.

## Data Availability

The data presented in this study are available on request from the corresponding author.
